# Internationalization, Hegemony, and Diversity: In Search of a New Vision for the Global Music Education Community

**DOI:** 10.1007/978-3-030-65617-1_14

**Published:** 2020-11-28

**Authors:** Alexandra Kertz-Welzel

**Affiliations:** 11 Queensland Conservatorium, Griffith University, Brisbane, QLD Australia; 12 Sibelius Academy, University of the Arts Helsinki, Helsinki, Finland; 13 Music Education and Music Therapy Department, Norwegian Academy of Music, Oslo, Norway; 14 Sydney Conservatorium of Music, University of Sydney, Sydney, NSW Australia; 15 Malmö Academy of Music, Lund University, Lund, Sweden; Ludwig-Maximilians-Universitaet, Munich, Germany

**Keywords:** Internationalization, Diversity, Educational transfer, Global music education community, Hegemony, Higher education, Global mindset

## Abstract

In higher education, internationalization is often seen as an exclusively positive development, even though there has been increased critique. This critique concerns a superficial understanding of internationalization as copying what globally successful universities do, thus ignoring local or national needs. But it is also related to the danger of confusing internationalization with Anglo-Americanization, in general and in various fields such as music education. Therefore, an investigation of what internationalization is with regard to music education and how it could look differently is much needed. This chapter critically analyzes internationalization in music education. At the core is the question of how internationalizing music education can be shaped in a way that overcomes hidden structures of hegemony. This chapter envisions a culturally sensitive internationalization of music education which acknowledges various teaching and research cultures. A framework, suggesting conceptual categories such as educational transfer or global knowledge production, can facilitate the formation of a united, yet diverse, global music education community. Additionally, selected concepts of community are presented that can be models for what a culturally sensitive international music education community could look like.

## Introduction

Internationalization, particularly in higher education, is often seen as an exclusively positive development. It offers universities opportunities for research collaborations and for proving their achievements in global rankings. Internationalization seems connected to endless possibilities for academic and financial success. But internationalization also has its downsides. By focusing on global dimensions, national, regional, or local aspects of universities are often neglected. Additionally, one teaching or research culture can dominate global discourses.

This chapter critically analyzes internationalization in music education, particularly in higher education. At the core is the question of how internationalizing music education can be realized in a way that overcomes hidden structures of hegemony. It envisions a culturally sensitive internationalization of music education which acknowledges various teaching and research cultures. A framework is presented, based on research findings from different fields. It suggests conceptual categories such as educational transfer or global knowledge production that need to be considered when aiming at the formation of a united, yet diverse, global music education community (Kertz-Welzel 2018). Furthermore, selected sociological concepts of community are presented to illustrate what such a community could look like.

The chapter starts with considerations about what internationalization is and what critical perspectives on it could mean, in general and regarding music education. The second part presents a framework with selected categories which concern music education especially in higher education.^1^ The following section develops the notion of a culturally sensitive global music education community. The final part offers perspectives for the future.^2^

## What Is Internationalization?

Even though internationalization is an omnipresent term, there is a lack of general research about this topic. Therefore, it is often not clear what it entails.^3^ Basically, internationalization has three different meanings. It stands for initiatives which go beyond national borders. It is connected to transnational political relationships. The term has likewise been used concerning a product which has been developed in one country and is adapted for the use in other countries (Kertz-Welzel 2018, pp. 3–5). Generally, internationalization is based on the notion of nation states.

Regarding higher education, internationalization describes initiatives going beyond national borders. It represents “the intentional process of integrating an international, intercultural, and global dimension into the purpose, functions, and delivery of post-secondary education … to make a meaningful contribution to society” (De Wit and Hunter 2015, p. 3). This indicates that internationalization is a goal-oriented process which deeply affects the very nature of universities. It can concern research and teaching partnerships or a global exchange of ideas. But it might likewise be about adopting new learning styles, thereby addressing the concerns of global students. It is also connected to universities’ mere general goals in terms of making meaningful contributions to society. Usually, there are four rationales for internationalization in terms of political, economic, social, and cultural as well as academic reasons, supporting the success of a university (De Wit 2011). They indicate internationalization’s multifaceted nature.

But there has also been critique regarding a superficial understanding. Knight (2011, 2012) and De Wit (2011) underline especially four problems of internationalization. First, internationalization should not be an end in itself; rather, it is supposed to aim toward fostering developing intercultural competencies and help preparing students for life and work in a global world. Second, internationalization concerns more than global rankings and partnerships; it affects the very nature of universities and should lead to changes regarding, for example, the teaching and research culture. Third, national differences and characteristics in higher education worldwide are important and meaningful in respective contexts. Therefore, internationalization should build on the local context and common ways of knowledge production, and not ignore them in favor of global standards; this includes implementing intercultural and global dimensions into the policies and programs of universities. Finally, on institutional and individual levels, the development of intercultural competencies is much needed. In general, successful internationalization addresses the specific needs of a respective university and connects it with universities in other countries. But the significance of local context means that there is no one-fits-all solution. Rather, internationalization might look different in each country and is certainly no easy process. The term glocalization has been applied to various processes in attending to some of these aspects, however has not been uncontroversial (Roudometof 2016).

Higher education and specific fields of research such as music education are thus in need of a positive, yet critical, vision of internationalization (Turner and Robson 2008). Brandenburg and De Wit (2011) even call for a “postinternationalization age.” It might be time to address the challenges internationalization poses. First, it is crucial to realize that internationalization is not a neutral term but is rather connected to specific cultures and knowledges. Aw (2017, p. xxii) states that “dominant paradigms in the conception of internationalization traditionally come from the English-speaking world and Western Europe.” She notes that internationalization is most often a one-sided process, for instance, from the Global North to the Global South, and not mutual. She states:

Internationalization involves knowledge exchange and transfer. However, the current practice is to privilege a form of knowledge originating from the North and flowing to the South. It is important that knowledge flows be multidirectional. Knowledge generation and dissemination need to be decolonized. (2017, p. xxii)

This demand could help redefining what internationalization in higher education is. Addressing issues of hegemony and marginalization is crucial, for example, regarding which knowledge is privileged. Aw (2017, p. xxii) suggests transforming what internationalization means toward having more “equitable policies and practices,” also more sustainable concepts of cooperation. A first step would be uncovering hidden hegemonic structures such as the dominance of the North (Aw 2017, p. xxii). This concerns higher education in general but also specific subject areas such as music education where the hegemony of Anglo-American music education has rarely been questioned.

Although internationalizing music education has been a topic addressed in music education research, it has hardly been investigated comprehensively. There also is seldom a distinction of internationalization in higher education and in schools (e.g., McCarthy 2012; Kertz-Welzel 2008). It would be interesting to identify commonalities and differences concerning internationalization in these two areas, particularly with regard to how they address diversity. McCarthy (2012, p. 57) generally argues for understanding music education from a global perspective, realizing its common purpose, but also acknowledging its national characteristics. She uses the metaphor of “global tapestry of music education,” illustrating the shared responsibility and challenges of music education worldwide. McCarthy identifies six challenges in global music education, related to music education as part of the public school curriculum: (1) the status of music education, (2) music education advocacy, (3) curriculum development and reform, (4) whose music is school music, (5) renewing the culture of pedagogy, (6) professional networks and forums for research. Most often, there have also been similar rationales for music education as part of the public school curriculum, for instance, nationalism and patriotism (Hebert and Kertz-Welzel 2012). Furthermore, educational transfer in terms of copying successful policies, strategies, or methods from other countries has been a well-known process in music education worldwide (Kertz-Welzel 2015). Methodologies such as Suzuki or Dalcroze are successful examples. While educational transfer is certainly not unproblematic, particularly regarding issues of hegemony when imperial powers force their models of schooling or teaching methods upon colonies (Philipps 2005), educational transfer is necessary to improve the quality of (music) education. But globally, it needs to be shaped in a more reflective way, taking issues of power into account. McCarthy (2012, p. 55) warns that “international perspectives in music education are founded on and dominated by narratives from Western countries and those influenced by the colonial presence of European countries.” There is indeed a need for raising awareness for geographical, geopolitical, and geolinguistic aspects of internationalization in music education. Acknowledging the diversity of music education and research cultures worldwide is therefore crucial for a culturally sensitive internationalization of music education in both universities and schools. A framework can facilitate this process.

## The Framework

A framework functions like a researcher’s map of the area investigated, providing a specific lens or perspective for scrutinizing a topic. It offers “a theoretical structure of categories and conceptual elements which can facilitate becoming a united and diverse global music education community” (Kertz-Welzel 2018, p. 10). A framework suggests various analytical categories and conceptual elements which arise from research findings in a respective field and can lead to new insights. Regarding music education, it can facilitate a culturally sensitive internationalization, for instance, concerning university programs preparing students for a global music education world or concerning international encounters (e.g., cooperation, conferences). It should not be something static but rather be expanded or revised through new research findings. The framework suggested here aims at overcoming the dominance of one music education tradition such as the Anglo-American one toward acknowledging the diversity of music education and research cultures worldwide. The notion of community regarding global community plays a crucial role in this process.

What could a culturally sensitive internationalization of music education look like? First, more knowledge about music education in various countries and internationalization will be paramount. This includes understanding what music education looks like worldwide and the impact internationalization had on it so far, e.g., regarding global exchange processes. This also includes how music education globally could be improved and internationalization shaped in a way supporting music education worldwide. Therefore, the framework offers selected categories addressing specific aspects of international music education such as educational transfer, international or comparative music education, and global knowledge production or the global mindset. They can be important points of reference and help shape internationalization in a culturally sensitive way.

Since music education is not a national field anymore, international and comparative music education can function as foundational research areas. Understanding music education as a global field of research means acknowledging the significance of educational transfer. This exchange of ideas has been going on at least since the eighteenth century when travelers from various countries came, for instance, to Switzerland or Germany, looking for the best instructional methods (Kertz-Welzel 2015). Since then, educational transfer in terms of copying successful strategies, methods, or policies from other countries has been most common. It has in recent years even been encouraged by international student assessments such as PISA (Program for International Student Assessment).^4^ Methodologies, for instance, the Orff-Schulwerk, represent global success stories of educational transfer. Even though they originated in a specific country such as Germany, they have been transferred to various countries worldwide, being adapted to new circumstances and respective musical traditions. The crucial issue, which educational transfer raises in view of comparative music education, is the fact that music education in various countries has already been globally connected and international for a long time. This relativizes the validity of comparative music education as a field and comparison as a method. Therefore, mapping the global flow of ideas might be more important for international music education than just being focused on comparing music education systems in different countries. Connecting comparative and international music education with analyzing the global flow of ideas in terms of educational transfer supports understanding music education as a global research area.

Furthermore, global knowledge production is a significant part of a framework facilitating a culturally sensitive internationalization of music education. No matter if the research is undertaken in a classroom or a concert hall, utilizes interviews or questionnaires, it contributes to the global knowledge in music education. However, even though research is going on worldwide, there is a significant impact of geographical, geopolitical, and geolinguistic factors. It matters where somebody conducts research, whether in a remote part of South America or in a well-known city of the United States. While knowledge is always generated in a specific context, it also needs to be generalizable and applicable to various circumstances in music education. Due to the international dominance of Anglo-American music education, it often seems to reviewers of journals, that knowledge which is not part of the Anglo-American music education world does not qualify to become global knowledge because it seems to be too locally bound (Kertz-Welzel 2018, pp. 64–73). Therefore, raising awareness of the politics of global knowledge production and critically analyzing them are important aspects of a culturally sensitive internationalization of music education.

Additionally, language is a crucial issue since English as lingua franca dominates international music education. Standards of good writing in English have long been accepted as international standards in music education. Not exactly following them, particularly regarding rhetorical choices, can mislead reviewers to conclude that authors do not only have a deficit in English but also in scientific thinking and research methods. Sociolinguistic research has frequently pointed out these issues (e.g., Mauranen 1993). There are, however, still problems with discriminating non-English native speakers in peer-reviewed journals as Lillis and Curry (2012) point out.^5^ The inability to make the most common rhetorical choices often leads reviewers to the conclusion that the scholarly competencies of authors whose native language is not English are limited. While it is necessary to have a sufficient language proficiency in English to be active in global music education research, more sensitivity regarding the problems of non-native English speakers is needed. Addressing the problems of research and publishing in a global world is a vital part of a culturally sensitive internationalization of music education.

A culturally sensitive global music education community is in need of culturally responsive music educators and scholars. Therefore, the global mindset is a useful concept. It summarizes the knowledge and abilities culturally sensitive people possess, for instance, regarding being open toward cultural diversity and to effectively communicate across cultures. It includes learning how to address cultural misunderstandings and conflicts, constantly learning and revising individual positions. The global mindset encompasses three different forms of capital in terms of psychological, intellectual, and social (Clapp-Smith et al. 2007). Regarding psychological capital, attributes such as curiosity, openness to new experiences, and cognitive flexibility are important. This likewise concerns being able to have a variety of perspectives on a situation, finding creative solutions that respect the values of different cultural contexts. Intellectual capital concerns having knowledge of different cultures, of globalization, of respective fields such as music education from an international perspective. The social capital of the global mindset describes the significance of relationships and networks for success in the global music education community. Gaining the different forms of capital the global mindset encompasses requires personal transformations which might not be easily accomplished. The global mindset is, however, an indispensable part of a culturally sensitive internationalization of music education.

The framework described above concerns different levels of internationalization in music education. By utilizing questions to investigate its current state in a specific country or region or regarding a respective topic, the framework can support the formation of a united, yet diverse, global music education community. Inquiring, for instance, which language or terminology is used in which circumstances, if there would be alternatives, or what terms are paramount can significantly facilitate internationalization and global encounters. This can include talking about the limits of translations, for instance, where we can easily understand each other and where not. Raising such issues opens up spaces for transformation and developing intercultural competence.

Certainly, the framework has many more areas than the ones mentioned above. Various sets of questions could be developed regarding research,^6^ music education policy^7^ or music education in general.^8^ These and many more queries can support a critical and culturally sensitive internationalization of music education, on a theoretical level. But they can also foster it in a more practical way in terms of facilitating collaborations between two or more countries. This helps to realize what unites and differentiates music education in various countries, in theory, practice, and research. Eventually, this process can lead to understanding international encounters as intercultural encounters. This means acknowledging similarities and differences between music education traditions but without eliminating them for the sake of oversimplification, because we are supposedly all the same, or overemphasizing dissimilarities, because we might not be alike at all. Saether and her colleagues (2012) describe this approach of intercultural encounters as “breaking the equilibrium and keeping the imbalance alive” (p. 367). It is about accepting diversity and not being too much focused on either similarities or differences in international music education. Understanding international encounters as intercultural encounters calls for intercultural understanding and a global mindset. This can facilitate the formation of a culturally sensitive global community.

## The Global Music Education Community Today

In the rhetorics on internationalization, the term “global community” is frequently used, even though it is not always clear what it entails (McCarthy 2012; Kertz-Welzel 2018). To support the formation of a united, yet diverse, global music education community, it is crucial to further investigate what the notion of community means in this context and to apply useful sociological concepts to music education.

“Community” generally describes a group of people who have something in common. They are united by specific values and ideas, sometimes even locality and language. Today, in view of globalization, communities are more flexible but also fragile, often not bound to a specific place or language anymore (Delanty 2018). Community has become a versatile concept which offers multifaceted perspectives for music education globally.

Music education worldwide might qualify as global community. Music educators share the same purpose regarding engaging people in music and supporting their musical learning. In some instances, they also face similar challenges regarding music education as a school subject (McCarthy 2012, p. 50). Often, they even have joint visions of what music education should accomplish, for example, regarding social change. Additionally, English is the common language in international music education. But what kind of community could the global music education community be?

Froehlich (2009, p. 92) characterizes the global music education community as a symbolic community. It has shared practices, values, and beliefs. They create a sense of belonging. To be a member in a symbolic community, identity formation is necessary which happens in different ways: at international conferences where the international presentation and discussion culture can be studied, including the rules of networking; by reading internationally important publications and getting to know significant researchers and discourses; in contact with individual scholars; and in seminars at universities where international students or professors discuss issues of global music education. Identity formation within the global music education community is an essential process and should be a topic in music education programs at universities worldwide. However, while music educators internationally might agree in many respects, for instance, regarding music education’s significance for children’s development, different opinions are welcome. Froehlich (2009, p. 94) states that “diversity can be shared and celebrated best if a sense of belonging has been established.” Shared basic beliefs provide a strong foundation for the global music education community, while it is enriched by a multiplicity of opinions and perspectives. In view of this diversity, the task of music educators should be to “work toward a sense of belonging across various geographical locales and for diverse social networks and groups” (Froehlich 2009, p. 94). The notion of symbolic community clearly indicates that a basic set of beliefs is sufficient to connect a variety of opinions and perspectives.

Aside from being a symbolic community, the global music education community could also be characterized as a cosmopolitan community. This notion is a useful model for addressing issues of globalization and internationalization, with community members of various nationalities and different perspectives who are united by basic beliefs (Delanty 2018, p. 179). A cosmopolitan community connects local and global perspectives and is de-territorialized, not restricted by space or time, flexible, but also fragile. At its core is the idea of humanity or the global civil society which has joint concerns such as climate change, refugees, and political populism – or everyone’s right to music and music education. Communication is crucial for cosmopolitan communities, facilitated by technology allowing members in various parts of the world to participate. Since cosmopolitan communities often represent something which concerns humanity at large, global music education certainly qualifies as a cosmopolitan community.

The notions of symbolic and cosmopolitan community provide useful visions for music education internationally. They help to understand how the global music education community can be shaped in a culturally sensitive way. While being united by basic beliefs, diversity is an integral part of successful communities. There is no need for the dominance of one music education or research culture. More intercultural dialogue might certainly be an important starting point to understand the current state of hegemony and diversity and to envision how the international music education community could look differently.

## Conclusion

Times of crises often highlight aspects which we usually overlook. Internationalization and the notion of global community might be such dimensions. The COVID-19 pandemic revealed that the world is closely connected and that some problems one country has are most likely to affect everyone. We are indeed a global community and more intensely linked than we ever thought. The global shutdown of universities and schools showed that we face similar challenges worldwide such as learning how to teach online. But the pandemic also led to a backlash of internationalization regarding an increased focus on national interests, for instance, resulting in closing borders. It exemplified that internationalization is still a work in progress and has so far not been accomplished in a sustainable way. There clearly is a need to critically reflect and refine what kind of global community we are and who we want to be, in general and in respective fields such as music education.

It will be the joint task of the global community to work on a culturally sensitive internationalization of music education. Only then can the challenges music education faces today and in the future be addressed. Developing a united, yet diverse, global music education community is the foundation for successful music education worldwide. This includes overcoming the hegemony of Anglo-American music education, particularly through investigations analyzing various music education and research cultures around the globe. This offers new ways of thinking and acting in music education, including new perspectives on global knowledge production. Canagarajah (2005) presents the following vision:

It is possible to develop a pluralistic mode of thinking where we celebrate different cultures and identities, and yet engage in projects common to our shared humanity. Breaking away from the history of constructing a globalized totality with uniform knowledge and hierarchical community, we should envision building a network of multiple centers that develop diversity as a universal project and encourage an actively negotiated epistemological tradition. (p. 20)

This is a call for embracing the diversity of music education and research cultures worldwide and to develop new ways of thinking and acting.^9^ It concerns creating a network of multiple research centers with groups of scholars conducting research on internationalization, developing ideas about how to implement diversity in international music education, while at the same time underlining what unites us globally. These groups could present what part of their own music education cultures might enrich international music education, including specific terminology unique to one tradition. They could investigate new ways of international exchange and cooperation, based on the vision of a united, yet diverse, global music education community. This might also concern critically investigating existing cooperation, their challenges, and opportunities. The Global Visions Project of the University of the Arts Helsinki (Johnson 2018) is an excellent starting point for such an endeavor, exemplifying how research and the practice of teacher education could be linked successfully in a culturally sensitive way. Additionally, more interdisciplinary research, readjusting the vision of diversity and music education to new global conditions, is much needed. The rise of nationalist and populist movements around the world, proclaiming their home country’s priority, can be dangerous for all attempts of internationalization. Addressing the challenges new political developments present to music education will be an important task. Therefore, internationalizing music education is also a political endeavor.

Another significant aspect is preparing students for being part of the global music education community. Thus, developing seminars on international and comparative music education, including educational transfer, is crucial, giving students the opportunity to learn more about music education theory and practice from a global perspective. This also concerns utilizing the international experiences many students bring to classes, not ignoring them in favor of Anglo-American standards. Internationalizing music education is a task for the global music education community. Each project and each scholar, student, or music teacher can be part of it supporting the vision of a united, yet diverse, global music education community.

## Footnotes

1 While the ideas presented are focused on higher education, they can easily be adapted to music education in schools, for instance, regarding the global mindset.

2 This chapter is based on research results presented in the publication “Globalizing music education” (Kertz-Welzel 2018) but also going beyond the scope of this book. It further develops the notion of community and is more closely related to recent research in higher education studies (Aw 2017).

3 There rarely is a comprehensive analysis of what internationalization means. Most studies investigate it as related to specific areas such as law (Varella 2014) or higher education (Knight 2012).

4 For more information, see: http://www.oecd.org/pisa/.

5 For more information about this topic regarding music education, see: Kertz-Welzel 2018, pp. 70–73.

6 What are important topics? What does the scholarly culture look like? Who are significant scholars? Is there research about internationalization and its problems in respective contexts? Is research conducted in one country more likely to be published than if it would have been conducted in another country?

7 What university model is implemented (e.g., British, German, American)? What role does educational transfer play, in higher education and regarding music education in schools? What is the role of music teacher education within the entire system of teacher education? What does internationalization mean in higher education? What opportunities and challenges are there? Who dominates international partnerships?

8 What are the goals of music education? What is the status of music education in schools? What are the most prominent teaching philosophies, methodologies or approaches? What challenges and opportunities does music education face in a respective country? What could we learn from each other?

9 The UNESCO document, *Rethinking education: towards a global common good?* (2015) also calls for embracing various kinds of knowledges and learning approaches.

## References

[CR1] Aw F, de Wit H (2017). Foreword. *The globalization of internationalization*.

[CR2] Brandenburg U, De Wit H (2011). The end of internationalization. International Higher Education.

[CR3] Canagarajah AS, Canagarajah AS (2005). Reconstructing local knowledge, reconfiguring language studies. *Reclaiming the local in language, policy and practice*.

[CR4] CIGE model for comprehensive internationalization. (2020). *American Council on Education*. http://www.acenet.edu/news-room/Pages/CIGE-Model-for-Comprehensive-Internationalization.aspx. Accessed 5 June 2020.

[CR5] Clapp-Smith R, Luthans F, Avolio BJ, Javidan M, Steers RM, Hitt MA (2007). The role of psychological capital in global mindset development. *The global mindset*.

[CR6] De Wit H (2011). Globalization and internationalization of higher education. Revista de Universidad Sociedad del Conocimiento.

[CR7] De Wit H, Hunter F (2015). The future of internationalization of higher education in Europe. International Higher Education.

[CR8] Delanty G (2018). *Community*.

[CR9] Froehlich H (2009). Music education and community: Reflections on ‘webs of interaction’ in school music. Action, Criticism and Theory for Music Education.

[CR10] Hebert D, Kertz-Welzel A (2012). *Patriotism and nationalism in music education*.

[CR11] Johnson, D. (Ed.), Westerlund, H., Sæther, E., Upadhyaya, P., Nagaraja, K., Dantchev, A., et al. (2018). *Confluence — Perspectives from an intercultural music exchange in Nepal* (Perspectives in Music and Music Education; No. 12). Lund: Musikhögskolan i Malmö, Lunds universitet.

[CR12] Kertz-Welzel A (2008). Music education in the 21st century: A comparison of German and American music education toward a new concept of global exchange. Music Education Research.

[CR13] Kertz-Welzel A (2015). Lessons from elsewhere? Comparative music education in times of globalization. Philosophy of Music Education Review.

[CR14] Kertz-Welzel A (2018). *Globalizing music education: A framework*.

[CR15] Knight J (2011). Five myths about internationalization. International Higher Education.

[CR16] Knight J (2012). Five truths about internationalization. International Higher Education.

[CR17] Lillis T, Curry MJ (2012). *Academic writing in a global context*.

[CR18] Mauranen A, Loefman L, Kurki-Suonio L, Pellinen S, Lehtonen J (1993). Cultural differences in academic discourse: Problems of a linguistic and cultural minority. *The competent intercultural communicator*.

[CR19] McCarthy M, McPherson GE, Welch GF (2012). International perspectives. *The Oxford handbook of music education*.

[CR20] Philipps D, Zajda J (2005). Policy borrowing in education: Frameworks for analysis. *International handbook on globalization, education and policy research*.

[CR21] Roudometof V (2016). *Glocalization: A critical introduction*.

[CR22] Saether E, Mbye A, Shayesteh R, McPherson GE, Welch GF (2012). Intercultural tensions and creativity in music. *The Oxford handbook of music education*.

[CR23] Turner Y, Robson S (2008). *Internationalizing the university*.

[CR24] United Nations Educational, Scientific, and Cultural Organization (UNESCO). (2015). *Rethinking education: Towards a global common good?* UNESCO Digital Library. http://unesdoc.unesco.org/images/0023/002325/232555e.pdf. Accessed 5 June 2020.

[CR25] Varella MD (2014). *Internationalization of law*.

